# Prime-Boost Vaccination with *Toxoplasma* Lysate Antigen, but Not with a Mixture of Recombinant Protein Antigens, Leads to Reduction of Brain Cyst Formation in BALB/c Mice

**DOI:** 10.1371/journal.pone.0126334

**Published:** 2015-05-26

**Authors:** Angelika Wagner, Irma Schabussova, Bärbel Ruttkowski, Roman Peschke, Józef Kur, Michael Kundi, Anja Joachim, Ursula Wiedermann

**Affiliations:** 1 Institute of Specific Prophylaxis and Tropical Medicine, Center for Pathophysiology, Infectiology & Immunology, Medical University of Vienna, Vienna, Austria; 2 Institute of Parasitology, Department of Pathobiology, University of Veterinary Medicine, Vienna, Austria; 3 Gdansk University of Technology, Faculty of Chemistry, Department of Microbiology, Gdansk, Poland; 4 Institute of Environmental Health, Medical University of Vienna, Vienna, Austria; 5 Department of Rheumatology & Inflammation Research, Institute of Medicine, University of Gothenburg, Gothenburg, Sweden; Charité, Campus Benjamin Franklin, GERMANY

## Abstract

**Introduction:**

Infection with the ubiquitous parasite *Toxoplasma gondii* is a threat for immunocompromised patients and pregnant women and effective immune-prophylaxis is still lacking.

**Methods:**

Here we tested a mixture of recombinant *T*. *gondii* antigens expressed in different developmental stages, i.e., SAG1, MAG1 and GRA7 (SMG), and a lysate derived from *T*. *gondii* tachyzoites (TLA) for prophylactic vaccination against cyst formation. Both vaccine formulations were applied systemically followed by an oral TLA-booster in BALB/c mice.

**Results:**

Systemic priming with SMG and oral TLA-booster did not show significant induction of protective immune responses. In contrast, systemic priming and oral booster with TLA induced higher levels of *Toxoplasma*-specific IgG, IgG1 and IgG2a in sera as well as high levels of *Toxoplasma*-specific IgG1 in small intestines. Furthermore, high levels of Toxoplasma-specific Th1-, Th17- and Th2-associated cytokines were only detected in restimulated splenocytes of TLA-vaccinated mice. Importantly, in mice orally infected with *T*. *gondii* oocysts, only TLA-vaccination and booster reduced brain cysts. Furthermore, sera from these mice reduced tachyzoites invasion of Vero cells *in vitro*, indicating that antibodies may play a critical role for protection against *Toxoplasma* infection. Additionally, supernatants from splenocyte cultures of TLA-vaccinated mice containing high levels of IFN-γ lead to substantial production of nitric oxide (NO) after incubation with macrophages *in vitro*. Since NO is involved in the control of parasite growth, the high levels of IFN-γ induced by vaccination with TLA may contribute to the protection against *T*. *gondii*.

**Conclusion:**

In conclusion, our data indicate that prime-boost approach with TLA, but not with the mixture of recombinant antigens SMG, induces effective humoral and cellular *Toxoplasma*-specific responses and leads to significant reduction of cerebral cysts, thereby presenting a viable strategy for further vaccine development against *T*. *gondii* infection.

## Introduction

*Toxoplasma gondii* is a ubiquitous, obligatory intracellular parasite. *T*. *gondii* oocysts are shed with cat faeces and may remain infectious in the environment for a long period of time. Generally, infection is acquired by ingesting raw meat products containing tissue cysts as well as via food or water contaminated with oocysts [1]. Contamination of drinking water with oocysts can lead to occasional outbreaks of toxoplasmosis [2, 3]. Even if infection with *T*. *gondii* may be asymptomatic in immunocompetent individuals, severe disease can develop in immunocompromised patients or foetuses of seronegative women with primary infection during pregnancy [4, 5].

Currently the only licenced *T*. *gondii* vaccine (Toxovax) is used in veterinary medicine. It contains live attenuated tachyzoites of the non-cyst forming S48 strain [6] and due to safety concerns is not suitable for human use. Therefore, research has focused on the development of safe inactivated vaccine formulations [7–9].

So far, the majority of animal studies testing vaccine candidates against *T*. *gondii* were performed in mouse strains which are lethally affected by *T*. *gondii* infection such as C57BL/6 mice. Furthermore, in these studies tachyzoites or tissue cysts were applied intraperitoneally, which does not mimic the route of infection in humans [10]. Therefore we previously established a mouse model of *T*. *gondii* infection where sporulated oocysts are administered orally to resistant BALB/c mice [11] hence most closely imitating natural *T*. *gondii* infection in humans. This model allows to evaluate vaccine efficacy on the basis of reduction of tissue cyst formation as major manifestation also of chronic infection in humans [12].

Crude extracts from the invasive tachyzoite stage (TLA) as well as several *T*. *gondii* surface and secreted protein antigens have been tested in different murine models in order to find an adequate vaccine candidate [7, 12, 13]. The best studied *T*. *gondii* antigen is SAG1, the major tachyzoite surface antigen and ligand for cell attachment which is crucial for cell invasion [14]. MAG1 is a matrix antigen of bradyzoites and tachyzoites [15]. GRA7 is expressed in all infectious stages of the parasite and is located in the dense granules and secreted at host cell entry [16]. Furthermore, GRA7 can be detected on the surface and in the cytoplasm of infected cells during the chronic infection. These three protein antigens are relevant in human infection as specific antibodies directed against SAG1, MAG1 and GRA7 can be detected in sera of *T*. *gondii* infected patients [15, 16]. It has previously been shown that immunisation of mice with each of the recombinant antigens, SAG1, MAG1 or GRA7 tested separately, can lead to prolonged survival in murine models of lethal *T*. *gondii* infection, even though sterilizing immunity was not achieved [17–20]. Due to the complexity of the *T*. *gondii* life cycle it seems that a monovalent vaccine with recombinant antigens is not sufficient for protection.

In this study we aimed to test two different vaccine antigen formulations, one based on a mixture of recombinant proteins derived from different developmental stages of the parasite (bradyzoites, tachyzoites and sporozoites comprised in oocysts), the other based on the whole extract of the tachyzoites, containing proteins but also non-protein components (carbohydrates, lipids etc.). Systemic priming with a mixture of the recombinant protein antigens SAG1, MAG1 and GRA7 (SMG) or tachyzoite lysate antigen followed by an oral booster with TLA was performed to test prevention of brain cysts formation after infection with *T*. *gondii* and to evaluate possible mechanisms of protection.

## Materials and Methods

### 2.1. Mice

Female BALB/c mice (6–8 weeks old) were purchased from the Research Institute for Laboratory Animal Breeding at the Centre of Biomedical Research, Medical University Vienna (Himberg, Austria). Experiments were approved by the Animal Experimentation Committee of the Medical University of Vienna and the University of Veterinary Medicine as well as by the Austrian Federal Ministry of Science and Research. (BMWF-68.205/0093-II/3b/2012 and BMWF-66.009/0213-II/3b/2010)

### 2.2. Parasites and antigens

*T*. *gondii* oocysts (laboratory strain “Hannover 1”) and tachyzoites of the *T*. *gondii* strain S-48 derived from Vero cell cultures were provided by the Institute of Parasitology, University of Veterinary Medicine, Vienna, Austria as previously described [11]. Tachyzoites were put through a syringe filter (5 μm, Minisart Sartorius, Goettingen, Germany) and further purified by discontinuous Percoll (GE Healthcare Biosciences AB, Uppsala, Sweden) density gradient centrifugation. For the preparation of *T*. *gondii* tachyzoite lysate antigen (TLA), tachyzoites were freeze-thawed in liquid nitrogen three times before protein quantification with BCA Protein Assay Reagent Kit (Pierce Peribo, Rockford, IL, USA). Recombinant antigens SAG1, MAG1 and GRA7 were expressed in *Escherichia coli* and purified according to the protocol described before [16].

### 2.3. Vaccination protocols and experimental setup

**Fig 1:**
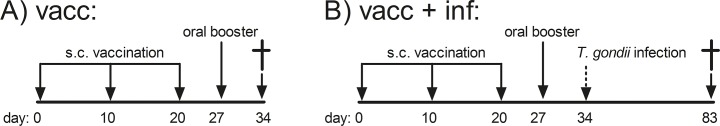
Experimental setup. (A) In the first experimental setup mice were subcutaneously (s.c.) vaccinated three times with SMG or TLA adjuvanted with GERBU in 10 day intervals. On day 27, an oral TLA booster adjuvanted with cholera toxin (CT) was administered in 3% NaHCO_3_ buffer and the experiment was terminated on day 34. Sham-vaccinated mice (sham) were sham-vaccinated with GERBU-PBS and sham-boosted with CT in 3% NaHCO_3_. (B) In the subsequent setup mice were systemically vaccinated and orally boosted as described in (A) before infection with 500 *T*. *gondii* oocysts in 3% NaHCO_3_ buffer on day 34 via gavage (SMG + inf; TLA + inf). Sham-vaccinated/infected mice (sham + inf) were sham-vaccinated with GERBU-PBS and sham-boosted with CT in 3% NaHCO_3_ before infection. Sham controls (sham + sham) were sham-vaccinated, sham-boosted and sham-infected receiving 3% NaHCO_3_ buffer on day 34. The experiment was terminated on day 83.

Mice were subcutaneously vaccinated three times in 10 day intervals with either a combination of the recombinant antigens SAG1, MAG1 and GRA7 (3 μg of each recombinant protein/mouse; referred to as SMG-vaccinated) or TLA (50 μg/mouse; referred to as TLA-vaccinated) adjuvanted with 50 μl GERBU (Gerbu, Heidelberg, Germany) (Fig 1A). GERBU belongs to a new generation of Freund´s complete adjuvant composed of glycopeptides derived from *Lactobacillus bulgaricus* cell walls (N-acetylglucosaminyl-N-acetylmuramyl-L-alanyl-D-isoglutamine) and cationic lipid solid nanoparticles. On day 27 both groups received an oral booster with TLA (50 μg/mouse) admixed to the classical mucosal adjuvant cholera toxin (CT), known as a potent inducer of mucosal and systemic humoral responses [21], (CT, 5 μg/mouse; Sigma-Aldrich, St Louis, MO, USA), which was administered in 300 μl of 3% NaHCO_3_ via gavage. Controls were sham-vaccinated with 50 μl GERBU-PBS subcutaneously three times and subsequently sham-boosted with 300 μl of 3% NaHCO_3_ with CT via gavage on day 27. On day 34 blood samples were taken and serum was obtained from coagulated blood samples after centrifugation at 1500 x *g* for 10 min and stored at -20°C until analysis. Thereafter mice were euthanized with isoflurane before spleens were excised (vacc: Fig 1A).

In the subsequent experimental setup, vaccination (as described above) was followed by infection with *T*. *gondii* oocysts, applied in 300 μl of 3% NaHCO_3_ via gavage, on day 34 (vacc + inf: Fig 1B). The infected controls (sham + inf) were sham-vaccinated, sham-boosted and thereafter orally infected as described above. In this setting sham controls (sham + sham) were sham-vaccinated and sham-boosted followed by application of 300 μl of 3% NaHCO_3_ via gavage on day 34. The experiment was terminated on day 83. Blood samples were collected on day 0, day 34 and on day 83 and serum samples prepared as described above. At day 83 mice were euthanized and spleens, small intestines and brains were excised for further analysis. All experiments were repeated twice and each experimental group consisted of 5 mice.

### 2.4. Gut lavage

At sacrifice, small intestines were excised and flushed with 2 ml PBS. Then the intestinal lumen was opened longitudinally and the tissue was stored in PBS supplemented with 1 x protease inhibitor (Complete Protease Inhibitor Cocktail Tablets, Roche, Mannheim, Germany) at -20°C until analysis. Upon thawing, 20% saponin solution was added and the samples were rotated overnight to elute antibodies from the mucosa before samples were centrifuged and supernatants were removed for antibody detection by ELISA.

### 2.5. Determination of *Toxoplasma*-specific antibody levels by ELISA

*Toxoplasma*-specific IgG, IgG1, IgG2a and IgA antibodies were measured in microtiter plates (Nunc, Roskilde, Denmark) coated with TLA at a dilution of 4 μg/ml in coating buffer (0.1 M carbonate-bicarbonate buffer). Sera (1:100 for IgG, IgG2a, IgA; 1:1000 for IgG1) or gut lavage fluids (undiluted for IgA; 1:10 for IgG1 and IgG2a) were incubated before adding peroxidase-conjugated rabbit-anti-mouse IgG for antigen-specific IgG detection (1:5000, Jackson Immuno Labs. Inc., West Grove, PA) or rat-anti-mouse IgG1, IgG2a or IgA (1:500, Pharmingen, San Diego, CA) followed by peroxidase-conjugated mouse-anti-rat IgG (1:2000).

### 2.6. *In vitro* stimulation of splenocytes

Spleens were homogenised to prepare single cell suspensions as previously described [11]. Cells (3 x 10^6^ per well in 48-well plates) were stimulated with TLA (1.5 μg/well) or incubated with medium at 37°C for 48 h. IFN-γ levels were determined in supernatants as described [22] and levels of IL-5, IL-6, IL-10 and IL-17 were measured using a Ready-SET-Go! ELISA kit (eBiocience, San Diego, CA, USA).

### 2.7. Quantification of *T*. *gondii*-specific DNA in brain tissue

Brain homogenates were prepared in 1 ml PBS by passing tissue through syringes with needles of decreasing diameters (14–23 G) as previously described [11]. Since tissue cysts may contain variable numbers of parasites, we established quantitative real time PCR as an accurate method of measuring the parasitic load in the brain tissue [23, 24]. Therefore 400 μl of brain homogenates were suspended in 360 μl ALT buffer (Quiagen, Valencia, CA) and 40 μl Proteinase K (Quiagen) before incubation at 56°C for 2 h. Thereafter DNA was extracted from a 50 μl aliquot of each brain lysate according to the protocol in the QIAmp DNA mini Kit (Quiagen). *T*. *gondii*-specific DNA was measured and quantified in samples of 30 ng of DNA derived from brain homogenates following the protocol of LightMix Kit *Toxoplasma gondii* (TIB MOLBIOL, Berlin, Germany) with a LightCycler instrument 1.2 (Roche, Basel, Switzerland) using the LightCycler FastStart kit. The DNA copies were calculated following the description of the LightMix Kit *Toxoplasma gondii* (TIB MOLBIOL).

### 2.8. Tachyzoite invasion and replication assay

To determine whether serum antibodies can mediate inhibition of tachyzoite cell invasion and replication, Vero cells suspended in culture media (complete medium; RPMI1640 with L-Glutamin; 4% FCS, 1% Pen/Strep; PAA Laboratories GmbH, Pasching, Austria) were seeded in 48-well plates (4 x 10^4^ cells in 500 μl medium per well). Tachyzoites (5 x 10^5^ of the S-48 strain) were mixed with pooled serum samples (diluted with complete medium at a concentration of 2.5% and sterile filtered with Millex Syringe Filters (0.22 μm, EMD Millipore Corporation, Billerica, MA, USA) and added to the cultured Vero cells. Sera were obtained from mice before (day 0) and after vaccination/oral booster (day 34). After 5 days of cultivation under standard conditions (5% CO_2_, 37°C) the medium including free tachyzoites was removed and the total number of tachyzoites/well was determined. Parasites were counted in aliquots of 10 μl/well using trypan blue for live staining. The assay was repeated three times. The reduction of the infection rates was calculated by extrapolation of counted tachyzoites after five days of cultivation to the standard curve in %, taking 5 x 10^5^ tachyzoites/well as 100% using EXCEL 2010 software. Results from sera of the non-vaccinated naïve control mice were considered as the reference value and defined as 100% and the reduction rates following incubation with sera of the TLA- and SMG-vaccinated and boosted mice were calculated as the percentage thereof.

### 2.9. Nitric oxide production

Murine macrophages (RAW 264.7 cell line a kind gift of Prof. Sylvia Knapp) were cultured in DMEM medium supplemented with 10% FCS (PAA, Pasching, Austria), penicillin and streptomycin (at 37°C, 5% CO_2_). For the assay, RAW 264.7 cells were plated at 1.5 x 10^5^/well in FCS-free DMEM medium. After overnight incubation, media were discarded and supernatants of TLA-restimulated splenocytes (collected on day 34), recombinant IFN-γ (eBioscience) or media alone were added. NO levels were evaluated in supernatants with Griess Reagent System (Promega, WI, USA) after 24 h of stimulation.

### 2.10. Statistics

Statistical analyses were performed by two-way ANOVA with group as a fixed factor and experiment as a random factor. Pairwise comparison between groups was done by Tukey's honest significant difference tests. Groups were considered significantly different if the two-sided *p* value obtained in the pair wise test was below 0.05. All statistical analysis was performed using Statistica 10.0 software (Statsoft, Tulsa, OK, USA).

## Results

### 3.1. Vaccination with the TLA lysate induced higher *Toxoplasma*-specific serum antibody levels than vaccination with the mixture of recombinant proteins

**Fig 2:**
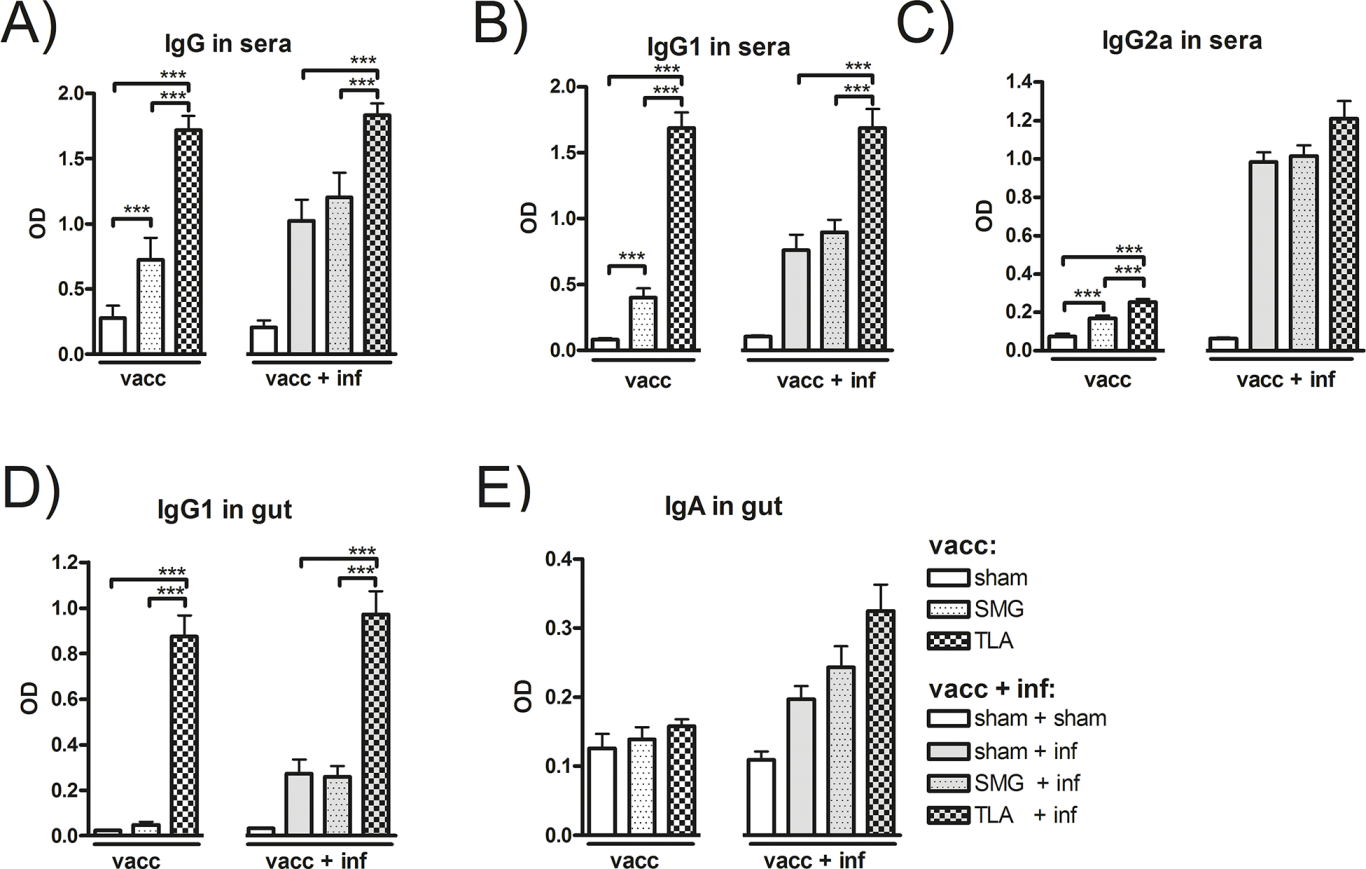
*Toxoplasma*-specific antibody levels in sera and gut. *Toxoplasma*-specific IgG (A), IgG1 (B) and IgG2a (C) antibody levels were measured in sera on day 34 (vacc) and day 83 (vacc + inf) by ELISA. Furthermore *Toxoplasma*-specific intestinal IgG1 (D) and IgA (E) levels were measured by ELISA in lavage fluids from the small intestines obtained at day 34 (vacc) and day 83 (vacc + inf). Results represent data of two independent experiments. Data are expressed as mean ± SEM. ***p < 0.001.

Vaccination with SMG and TLA followed by an oral TLA-booster led to production of *Toxoplasma*-specific serum antibodies in both treatment groups. Comparing these groups and sham vaccinated mice (Fig 2A–2C; vac), highest *Toxoplasma*-specific IgG, IgG1 and IgG2a levels were detected in TLA-vaccinated mice. Upon infection of TLA-vaccinated mice, IgG and IgG1 antibody levels remained at high levels, whereas IgG2a levels even increased after to infection (Fig 2A–2C; vacc + inf). In SMG-vaccinated mice however, the IgG and IgG1 antibody levels after vaccination (vacc) were significantly lower compared to TLA-vaccinated mice (Fig 2A and 2B; vacc). Though antibody levels increased following infection (vacc + inf) they still remained lower than in the TLA-vaccinated mice.

### 3.2. Vaccination with TLA, but not with the protein mixture SMG, led to elevated *Toxoplasma*-specific antibodies in the gut

Since the vaccination protocol included an oral booster with TLA adjuvanted with CT, we evaluated mucosal antibody responses in the gut. Vaccination with TLA elicited significantly higher *Toxoplasma*-specific IgG1 in the gut compared to the SMG- or the sham-vaccinated mice (Fig 2D: vacc) and these levels were maintained also after infection (Fig 2D: vacc + inf). Vaccination with SMG did not lead to significantly increased *Toxoplasma*-specific IgG1production in the gut compared to sham-vaccinated mice, even though after infection, moderate *Toxoplasma*-specific IgG1, were measured. In contrast, *Toxoplasma*-specific IgA levels did not differ between the groups after systemic vaccination (Fig 2E: vacc) though levels slightly increased after infection (Fig 2D: vacc + inf), showing the highest rise in the TLA-vaccinated group.

### 3.3. Vaccination with TLA, but not with SMG, led to enhanced cytokine production in restimulated splenocytes *in vitro*

**Fig 3:**
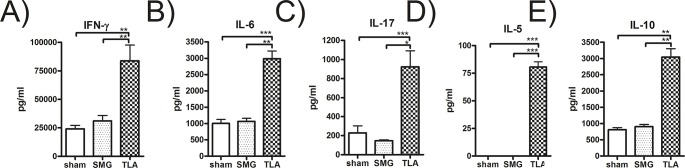
Cytokine production of splenocytes stimulated with TLA *in vitro*. After systemic vaccination and oral booster splenocytes were excised on day 34 and single cell suspensions were prepared and stimulated with TLA *in vitro* before cytokine levels were determined by ELISA. Results represent data of two independent experiments. Data are expressed as mean ±SEM. * *p* < 0.05; ** *p* < 0.01; *** *p*< 0.001.

Splenocytes of TLA-vaccinated, but not SMG-vaccinated mice showed a significantly higher production of IFN-γ, IL-6, IL-17, IL-5 and IL-10 after restimulation with TLA compared to sham controls (Fig 3A–3E). Also after infection, these cytokine levels were significantly increased in spleen cell cultures of the TLA-vaccinated compared to the sham-vaccinated mice (Table 1)—at this time point also SMG-vaccinated and infected mice showed increased levels of IL-17 and IL-10 compared to sham-vaccinated controls (Table 1).

**Table 1:** Cytokine production of splenocytes stimulated with TLA *in vitro*.

day 83	IFN-γ ± SEM (ng/ml)	IL-6± SEM (pg/ml)	IL-17± SEM (pg/ml)	IL-5± SEM (pg/ml)	IL-10± SEM (pg/ml)
**sham**	59.7 ±26.6	630.5 ±188.3	227.3 ±48.7	0.0 ±0.0	740.9 ±172.7 ^aaa^
**inf**	127.6 ±22.1	1529.6 ±411.2	213.0 ±48.3	17.1 ±12.1	2698.3 ±±298.3
**SMG**	169.76 ±24.7 ^b^	1586.5 ±322.0	428.3 ±97.2 ^a^ ^,^ ^bb^	22.3 ±1.5	5333.4 ±700.7 ^a^
**TLA**	178.8 ±19.3 ^a^	2036.7 ±612.5 ^aa^	1938.4 ±548.5 ^aa^	59.4 ±24.6	6766.8 ±424.0 ^aaa^

Spleens were excised on day 83 (after vaccination and infection) and single cell suspensions were prepared and stimulated with TLA *in vitro*. Results represent data two independent experiments. Data are expressed as mean ±SEM.

^a^comparison to sham-vaccinated and infected mice

^a^ *p* < 0.05

^aa^ *p* < 0.01

^aaa^ *p* < 0.001.

^b^comparison to TLA-vaccinated and infected mice

^b^ *p* < 0.05

^bb^ *p* < 0.01.

### 3.4. Significant reduction of *T*. *gondii*-specific DNA in brain tissue was observed only in TLA-vaccinated mice

**Fig 4:**
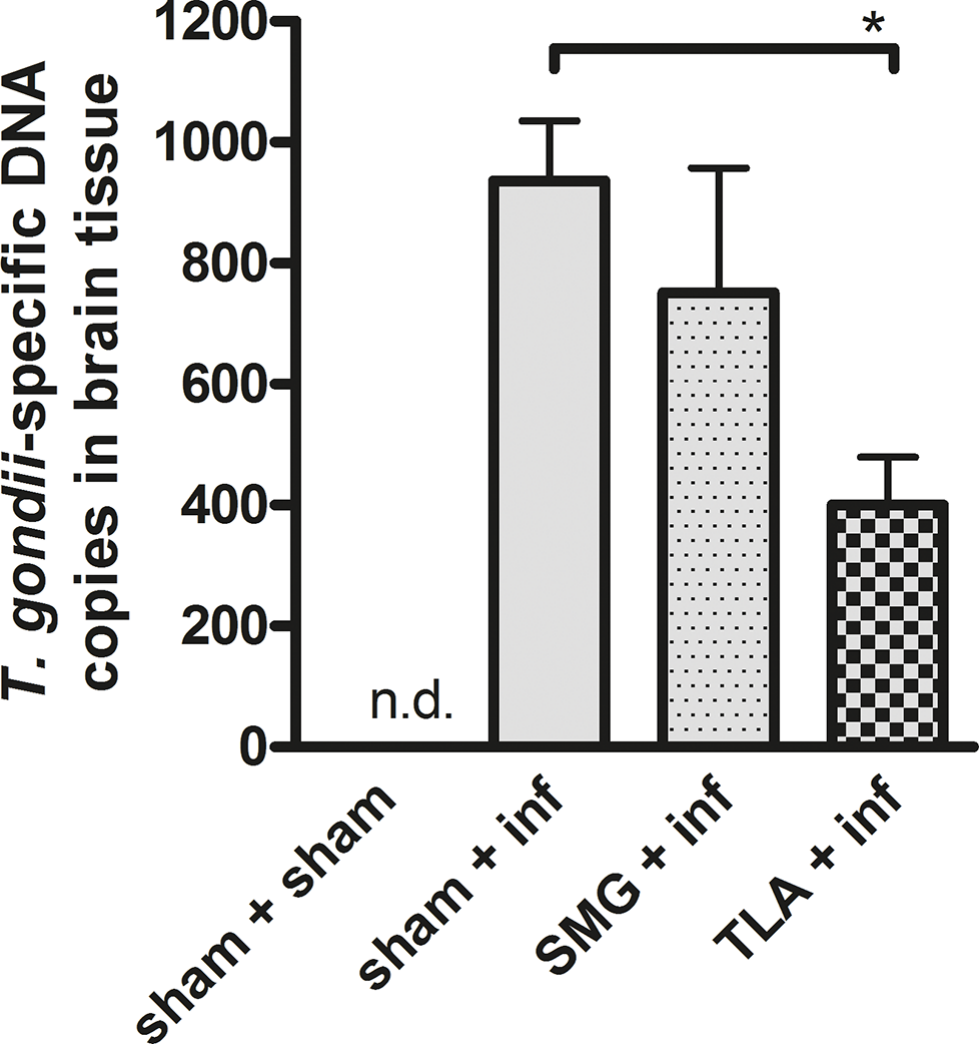
*T*. *gondii*-specific DNA copies in brain tissue. In systemically vaccinated and orally boosted brains were excised after infection on day 83. The number of *T*. *gondii*-specific DNA copies was determined in homogenised brain tissue by quantitative Real-time RT PCR. Bars represent mean ± SEM. Results represent data of two independent experiments. Data are expressed as mean ±SEM. *p < 0.05. (ND not detectable).

In order to obtain reliable data on cerebral parasite burden, PCR-based measurement of *T*. *gondii-* specific DNA copies in brain homogenates was performed. These measurements revealed that *T*. *gondii*-specific DNA copies in brain homogenates were significantly reduced only in the TLA- vaccinated and infected mice but not in the SMG-vaccinated infected animals (Fig 4).

### 3.5. Significantly higher reduction of tachyzoite invasion and replication *in vitro* by sera from TLA-vaccinated than from SMG-vaccinated mice

**Fig 5:**
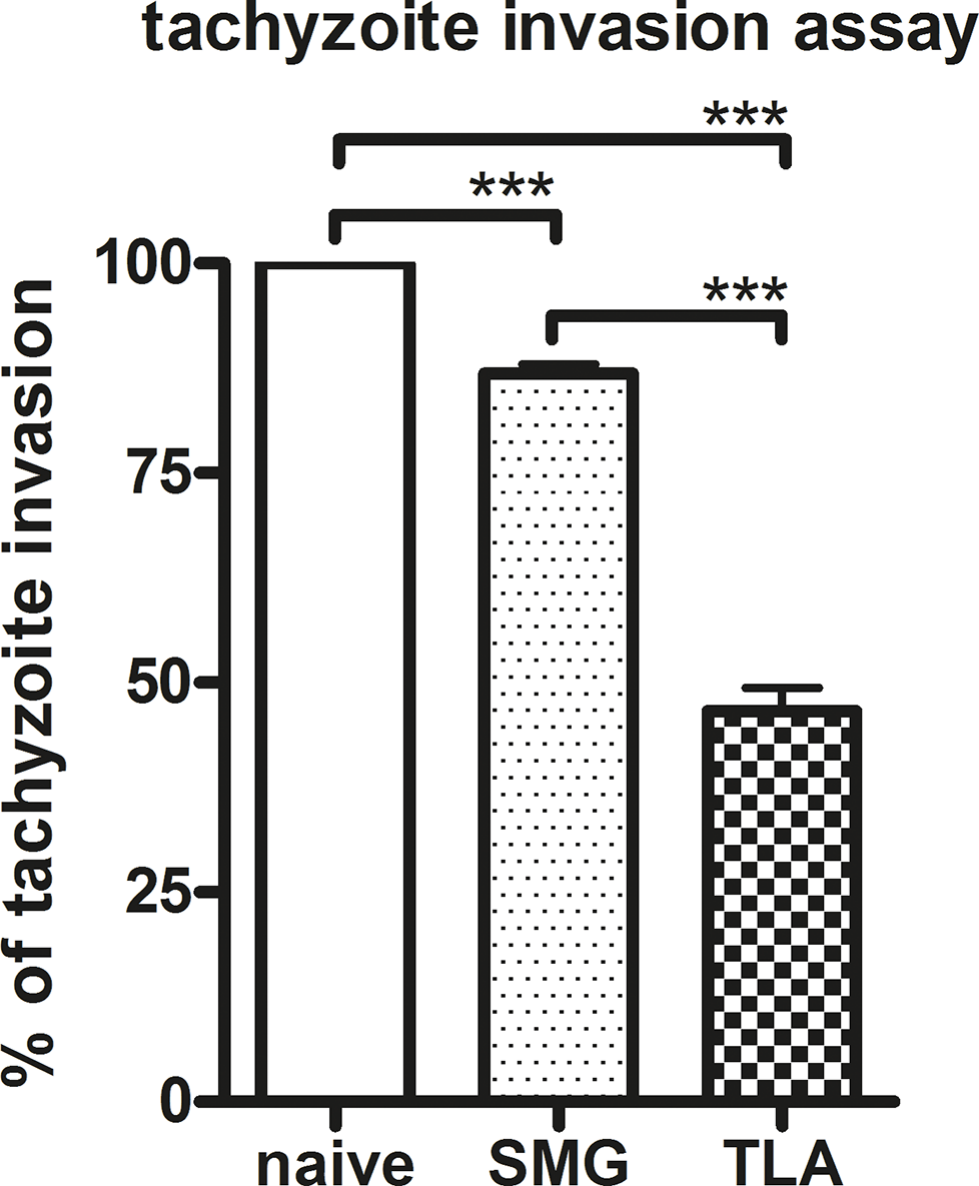
Tachyzoite invasion and replication assay. Tachyzoites were pre-incubated with sera from naïve mice collected on day 0, or with sera collected from SMG-vaccinated or TLA-vaccinated and orally boosted mice on day 34 (i.e. prior to infection). Bars represent mean percentage of reduced tachyzoite invasion and replication of Vero cells after incubation. *In vitro* assays were performed with pooled serum samples (n = 5) repeated three times. Results represent data of three independent experiments. Data are expressed as mean ± SEM. ***p < 0.001.

Since reduction of *Toxoplasma*-specific DNA copies in brains was accompanied by increased *Toxoplasma*-specific antibody levels, sera of these mice were applied in a *T*. *gondii* tachyzoite invasion and replication assay using Vero cells. Incubation of tachyzoites with sera of TLA-vaccinated mice led to significantly higher reduction of tachyzoite invasion and replication in Vero cells than SMG-vaccinated mice (53% TLA versus 13% SMG) (Fig 5).

### 3.6. Increased NO production in macrophages after stimulation with supernatants of spleen cell cultures from TLA-vaccinated mice

**Fig 6:**
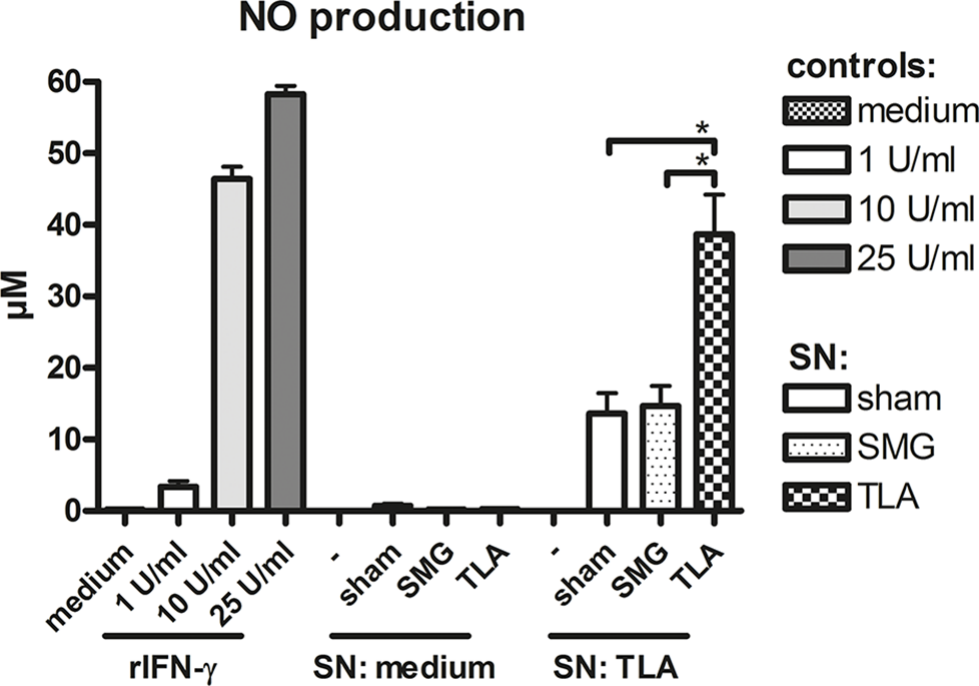
Nitric oxide production. Murine macrophages (RAW264.7) were incubated for 24h with supernatants (SN) of TLA-restimulated (TLA) or unstimulated (medium) splenocytes obtained on day 34. Different concentrations of recombinant IFN-γ (rIFN-γ) or medium alone served as controls. NO concentrations were measured with a Griess assay. Results represent data of three independent experiments. Data are expressed as mean ± SEM. *p < 0.05.

It has been described that IFN-γ can induce NO production by macrophages thereby mediating restriction of parasite replication within the cells [25]. Since highest levels of IFN-γ were detected in supernatants of restimulated splenocytes (day 34) from TLA-vaccinated mice, we tested whether the IFN-γ containing supernatants could stimulate NO production in a murine macrophage cell line (RAW 264.7). Indeed, the highest NO production was induced after incubation of RAW cells with supernatants of TLA-vaccinated mice (Fig 6). In contrast, supernatants derived from SMG-vaccinated mice (containing significantly less IFN-γ) did not increase NO levels compared to sham-vaccinated and medium stimulated controls.

## Discussion

Here we tested two different vaccine antigen formulations in order to prevent oral *T*. *gondii* infection: (a) a tachyzoite lysate antigen (TLA), and (b) a mixture of three recombinant *T*. *gondii* antigens SAG1, MAG1 and GRA7 (SMG). The vaccination protocol combined systemic and oral vaccination in a prime-boost schedule in order to induce immune responses at the primary site of parasite entrance, i.e. the gut, as well as at sites of systemic replication and cyst formation. Along these lines, a combination of systemic and mucosal administration of vaccine antigens has been shown to enhance both systemic antibody and cytokine responses as well as local/mucosal immune responses in a murine model [26], which however has not been tested so far in a vaccination model against *T*. *gondii* infection.

Our results show that systemic and oral vaccination with TLA led to high levels of *Toxoplasma*-specific IgG antibodies, particularly IgG1, in serum and small intestines (Fig 2) as well as high levels of cytokines in restimulated splenocytes (Fig 3, Table 1). The importance of combining the systemic and oral immunisation route to induce protective immune responses was seen after we have conducted a series of pilot experiments where neither systemic immunisation alone nor sole oral applications of the vaccine antigens led to significant immune responses or protection against *T*. *gondii* infection and cyst formation (data not shown).

In TLA-vaccinated and infected mice, the brain cyst burden was significantly reduced suggesting that both, the strong humoral and cellular responses induced by TLA-vaccination correlated with protection (Fig 4). The contribution of antibodies to the defence against pathogens has been described to be mediated by different mechanisms, including inhibition of adhesion to host cells [27]. Thus, to test whether the antibodies induced in our model can prevent parasite adhesion and thereby infection, we performed a tachyzoite invasion assay demonstrating that sera derived from TLA-vaccinated mice, but not from SMG-vaccinated mice, reduced invasion and replication of tachyzoites in Vero cells *in vitro*. These results are in agreement with other studies showing that *Toxoplasma*-specific antibodies can prevent invasion/infection of enterocytes *in vitro* [28–30]. Even though the test was performed with whole serum samples and not with isolated antibodies, it seems reasonable to attribute the protection to the high *Toxoplasma*-specific antibody titres, since sera from naïve/sham immunized mice did not show any inhibiting effect. Moreover, vaccine-induced *Toxoplasma*-specific antibodies have been previously shown to be involved in the protection *in vivo*; Sayles et al showed that *T*. *gondii* infection was lethal in B-cell deficient (μMT) mice unless *T*. *gondii*-immune sera were substituted leading to prolongation of their survival [30]. In our study, the highest level of *Toxoplasma*-specific antibodies in sera as well as in gut lavage fluids belonged to the IgG subclass, in particular the IgG1 isotypes, which might thus be responsible subclass to hinder invasion.

Apart from antibodies, another important line of parasite defence has been attributed to cytokines such as IFN-γ, IL-17 and IL-6, all of them playing a role in coordinating the innate and adaptive defence mechanisms of the host during infection with *T*. *gondii*. It is well established that IFN-γ is important to promote Th1-directed adaptive immune responses [31] and to control parasite replication during chronic infection [32, 33] via IFN-γ mediated NO synthesis in different host cells such as macrophages [34]. IL-6 has been shown to effectively induce IL-17 production during *T*. *gondii* infection [35] and IL-17 is required for survival, since its absence results in increased parasite burden and mortality associated with a lack of polymorphonuclear cells migrating to the site of parasite entry and replication as demonstrated [36]. Along these lines, we detected high levels of IL-6, IL-17 and IFN-γ secretion in restimulated splenocytes of TLA-vaccinated mice. Moreover, supernatants with high IFN-γ were able to induce NO synthesis in a murine macrophage cell line *in vitro*. These results are suggestive that the vaccine-induced IFN-γ in our model could also trigger NO production *in vivo* thereby improving host resistance to the parasite.

Th2 cytokines such as IL-5, and IL-10 were also detected in splenocyte cultures of TLA-vaccinated mice upon restimulation. In particular, IL-10 is important to counter-balance the proinflammatory immune responses to avoid detrimental effects for the host [37], while IL-5 has been described to mediate protective properties as *T*. *gondii* infection is lethal in IL-5 KO mice [38].

The central finding in this study was that all these preventive responses were only achieved with the TLA extract but not with the recombinant protein mixture SMG. TLA is a mixture of diverse antigens that contain protein, carbohydrate and lipid components [39–41], having the potential to activate innate and adaptive immune responses via different pathways in contrast to pure protein formulations. The observation that vaccination with a whole cell extract can be more efficient than with selected protein antigens has been demonstrated for certain human vaccines. In this respect, recent studies revealed that the former whole cell vaccine against *Bordetella pertussis* confers a broader and longer lasting protection (at cost of higher reactogenicity, though) compared to the currently used acellular vaccine containing 2–3 selected purified protein antigens [42, 43]. This might be explained by the fact that only the whole cell vaccine contains pathogen-associated molecular patterns that via Toll-like receptor (TLR) engagement stimulates innate and adaptive immune responses such as IgG antibodies, IFN-γ and IL-17 cytokine production thereby establishing broad protective responses [42]. Along these lines our preliminary data show that stimulation of murine bone marrow derived dendritic cells and splenocytes with TLA and heat inactivated TLA (resulting in denaturation of proteins) led to comparable amounts of proinflammatory cytokine production *in vitro*. This indicates that not only proteins but also carbohydrates and lipids may add to the induction of protective immune responses in our model.

Taken together, systemic vaccination with TLA plus an oral booster with TLA resulted in a significantly reduced parasite burden in brains of infected mice. Reduction of cerebral cysts correlated with high levels of systemic and mucosal *Toxoplasma*-specific antibodies and cytokine production such as IFN-γ known to induce NO-mediated defence mechanisms.

Our data indicate that efficient vaccination against *T*. *gondii* infection requires activation of both innate and adaptive immune responses. Since the use of crude extracts bears the risk of higher reactogenicity an improved vaccine strategy could include the careful selection of the vaccine antigen(s) in combination with adjuvants that can adequately stimulate innate and adaptive immune responses comparable to whole microbial extracts.
